# High occurrence of *Blastocystis* sp. subtypes 1–3 and *Giardia intestinalis* assemblage B among patients in Zanzibar, Tanzania

**DOI:** 10.1186/s13071-016-1637-8

**Published:** 2016-06-29

**Authors:** Joakim Forsell, Margareta Granlund, Linn Samuelsson, Satu Koskiniemi, Helén Edebro, Birgitta Evengård

**Affiliations:** Division of Clinical Bacteriology, Department of Clinical Microbiology, Umeå University, SE-901 87 Umeå, Sweden; Division of Infectious Diseases, Department of Clinical Microbiology, Umeå University, SE-901 87 Umeå, Sweden

**Keywords:** Zanzibar, Tanzania, *Blastocystis*, Subtype, *Giardia*, Assemblage, Real-time PCR, Genotyping

## Abstract

**Background:**

*Blastocystis* is a common intestinal parasite with worldwide distribution but the distribution of *Blastocystis* and its subtypes in East Africa is largely unknown. In this study, we investigate the distribution of *Blastocystis* subtypes in Zanzibar, Tanzania and report the prevalence of intestinal parasites using both molecular methods and microscopy.

**Methods:**

Stool samples were collected from both diarrhoeic and non-diarrhoeic outpatients in Zanzibar. In addition to microscopy, real-time PCR for *Blastocystis*, *Entamoeba histolytica* and *E. dispar*, *Giardia intestinalis*, *Cryptosporidium* spp., and *Dientamoeba fragilis* was used. *Blastocystis* subtypes were determined by a conventional PCR followed by partial sequencing of the SSU-rRNA gene. Genetic assemblages of *Giardia* were determined by PCR with assemblage specific primers.

**Results:**

Intestinal parasites were detected in 85 % of the 174 participants, with two or more parasites present in 56 %. *Blastocystis* sp. and *Giardia intestinalis* were the most common parasites, identified by PCR in 61 and 53 % of the stool samples respectively, but no correlation between carriage of *Blastocystis* and *Giardia* was found*.* The *Blastocystis* subtype distribution was ST1 34.0 %, ST2 26.4 %, ST3 25.5 %, ST7 0.9 %, and 13.2 % were positive only by qPCR (non-typable). The *Giardia* genetic assemblages identified were A 6.5 %, B 85 %, A + B 4.3 %, and non-typable 4.3 %. The detection rate with microscopy was substantially lower than with PCR, 20 % for *Blastocystis* and 13.8 % for *Giardia.* The prevalence of *Blastocystis* increased significantly with age while *Giardia* was most prevalent in children two to five years old. No correlation between diarrhoea and the identification of *Giardia*, *Blastocystis*, or their respective genetic subtypes could be shown and, as a possible indication of parasite load, the mean cycle threshold values in the qPCR for *Giardia* were equal in diarrhoeic and non-diarrhoeic patients.

**Conclusions:**

Carriage of intestinal parasites was very common in the studied population in Zanzibar. The most commonly detected parasites, *Blastocystis* and *Giardia,* had different age distributions, possibly indicating differences in transmission routes, immunity, and/or other host factors for these two species. In the *Blastocystis* subtype analysis ST1-3 were common, but ST4, a subtype quite common in Europe, was completely absent, corroborating the geographical differences in subtype distributions previously reported.

## Background

Even though most intestinal parasites are encountered worldwide, they are more common in low-income countries with tropical or subtropical climate and a low grade of sanitation. They are most often spread through faecal-oral transmission routes by ingestion of contaminated food or water. In the last two decades, tools for molecular detection and genetic characterisation of intestinal parasites have been developed, which has improved the ability to diagnose these organisms in stool samples. In addition, genotyping of the parasites has led to several advances, one important example being the separation of morphologically identical *Entamoeba* cysts into the pathogenic *Entamoeba histolytica* and the nonpathogenic *Entamoeba dispar* [1]. An increasing amount of genotype data is being collected for several different species of intestinal parasites which is fundamental to deepen the understanding of pathogenicity, host specificity and transmission patterns of these organisms.

The anaerobic unicellular eukaryote *Blastocystis* sp. is a unique organism among the intestinal parasites, belonging to the phylum Stramenopiles [2]. It frequently inhabits the gastrointestinal tract of both humans and a large variety of animals [3, 4]. In intestinal parasitological surveys in humans, it is often the most commonly found organism and the prevalence ranges from 7 to 20 % of examined individuals in developed countries [5–7] to 30–60 % [8], or even 100 % [9], in rural areas in developing countries. The presence of *Blastocystis* in the intestine has been associated with diarrhoea, flatulence, bloating and other irritable bowel syndrome (IBS)-like symptoms. However, the pathogenicity of the organism is debated, mainly because a high carrier-rate has also been reported in asymptomatic individuals. Differences in host susceptibility, host intestinal microbiota and/or different pathogenic potential of the different genetic subtypes of *Blastocystis* has been put forth as possible explanations to these differences in observed pathogenicity [10, 11]. There is a high degree of genetic diversity among *Blastocystis* isolates and the genetic subtypes are named ST1-17 [4, 12–15]. ST1-9 has so far been found in humans, with a majority belonging to ST1-4 [16]. In Africa, the prevalence of *Blastocystis* subtypes has been studied in the northern and western parts of the continent, namely Egypt [17–20], Libya [16, 21], Senegal [9], Liberia and Nigeria [16], while data from East Africa is sparse. A study from Petrášová et al. [22] described the *Blastocystis* subtypes found in non-human primates, as well as in six human researchers, in Rubondo Island in the northwest part of Tanzania. Other than that, data on subtype distributions in humans in this region are, to the best of our knowledge, lacking. In this study we have examined the prevalence and subtype distribution of *Blastocystis* sp. in health care seekers in Jambiani, a coastal village on the island Zanzibar, Tanzania. We have also investigated the general prevalence of intestinal parasites in this population using both microscopy and, for selected parasites, real time PCR.

## Methods

### Sample collection and ethical approval

The presence of intestinal parasites was investigated in people living in Jambiani village, Zanzibar, Tanzania, a small village on the southeast coast of the main island Unguja. The majority of the population is Muslim and engaged in subsistence farming, seaweed farming, fishing and/or tourism. Many of the people live under poor sanitary conditions, with a majority relying on local wells for water supply. For a number of years in the early 2010s clinical stool samples from patients seeking care at an out-patient health clinic in Jambiani, were analyzed for parasites at the Clinical Microbiology laboratory, Umeå, Sweden due to a personal contract between the local health clinic authorities and one of the researchers (BE). Two samples per patient, one stored in L2-buffer containing guanidine thiocyanate [23] and one stored in sodium acetate-acetic acid-formalin fixative (SAF), were sent numbered from anonymised patients for molecular and microscopic parasite detection. In consultation with the medical staff at the health clinic, treatment was given to patients that were diagnosed with what was interpreted as clinically relevant intestinal parasites. All faecal samples were stored at +4 °C prior to parasite detection and DNA used for molecular detection was kept frozen at -70 °C. In this study, consecutively collected samples were further examined with subtyping of *Blastocystis* sp. and *Giardia intestinalis*. The study was approved by the regional ethical board at Umeå University, Sweden (Dnr 2012-406-31M). The samples investigated could be divided into two groups, one from patients zero to ten years old irrespective of the presence of diarrhoea (*n* = 108) and patients more than ten years old mainly with diarrhoea (62/66).

### Microscopy

Samples stored in SAF were concentrated by the formol-ether-concentration technique (FECT) [24] followed by parasite detection by light microscopy. Iodine staining was used to detect helminth ova and protozoan cysts, and a modified Ziehl-Neelsen acid-fast staining, using carbol fuchsin, decolorization with hydrochloric acid and counterstaining with malachite green, was used to detect coccidian oocysts of genera *Cryptosporidium*, *Cyclospora* and *Cystoisospora*.

### DNA extraction

DNA was extracted from samples stored in L2 buffer (guanidine-thiocyanate 0.96 g/ml dissolved in 0.1 M Tris, pH 6.4) using a method previously developed in our laboratory [23]. Briefly, 150–200 mg faeces was collected with a flocked nylon swab (ESwab, Copan) and transferred to 1 ml of Amies medium, 400 μl of this suspension was transferred to a tube with Lysing matrix E that contains beads (MP Biomedicals, Nordic Biolabs AB, Täby, Sweden). Bead beating was performed with a FastPrep-24 Instrument at 6 m/s for 40s (MP Biomedicals). Following centrifugation, 250 μl lysate was diluted with 250 μl nuclease free (NF)-water and transferred to the automatic instrument Arrow and run with Arrow stool DNA kit according to the manufacturer’s instructions (NordDiag, Oslo, Norway). DNA was eluted in 100 μl elution buffer and stored in −70 °C before PCR amplification.

### Real-time PCR (qPCR) assays to detect *Entamoeba histolytica*, *Entamoeba dispar*, *Giardia intestinalis*, *Cryptosporidium* spp. and *Dientamoeba fragilis*

*Entamoeba histolytica* and *Entamoeba dispar* were detected by a previously described duplex qPCR assay with the genus specific primer pair Ehd-239 F and Ehd-88R and the species specific probes Histolytica 96 T and Dispar 96 T [25]. The reaction mixture contained 12.5 μl TaqMan Universal PCR Master Mix (Applied Biosystems, Life Technologies, Stockholm, Sweden), primers Ehd-239 F (5′-ATT GTC GTG GCA TCC TAA CTC A-3′) and Ehd-88R (5′-GCG GAC GGC TCA TTA TAA CA-3′) each at 176 nM, probes histolytica 96 T (VIC 5′-TCA TTG AAT GAA TTG GCC ATT T-3′-MGBNFQ) and dispar 96 T (FAM 5′-TTA CTT ACA TAA ATT GGC CAC TTT G-3′-MGBNFQ) each at 224 nM, 5 μl DNA template, and nuclease free (NF)-water to a final volume of 25 μl. A multiplex qPCR assay developed at the Dutch National Institute for Public Health and the Environment (RIVM) using previously published primers and probes was used to detect *Giardia intestinalis* [26], *Dientamoeba fragilis* [27] and *Cryptosporidium* spp. [28]. The assay was modified from a Lightcycler protocol to a Taqman protocol with different fluorophores and quenchers conjugated to the probes. Our reaction mixture consisted of 12.5 μl TaqMan Universal PCR Master Mix (Applied Biosystems), primers Giardia 80 F (5′-GAC GGC TCA GGA CAA CGG TT-3′) and Giardia 127R (5′-TTG CCA GCG GTG TCC G-3′) each at 200 nM, probe Giardia T (FAM-5′-CCC GCG GCG GTC CCT GCT AG-3′-BHQ1) at 40 nM, primers CrF (5′-CGC TTC TCT AGC CTT TCA TGA-3′) and CrR (5′-CTT CAC GTG TGT TTG CCA AT-3′) each at 600 nM, probe Crypto (CY5-5′-CCA ATC ACA GAA TCA TCA GAA TCG ACT GGT ATC-3′-BHQ2) at 200 nM, primers Df-124 F (5′-CAA CGG ATG TCT TGG CTC TTT A-3′) and Df-221R (5′-TGC ATT CAA AGA TCG AAC TTA TCA C-3′) each at 560 nM, probe Df-172revT (VIC-5′-CAA TTC TAG CCG CTT AT-3′-BHQ1) at 200 nM, 0.5 μl BSA 8 mg/ml, 5 μl DNA template and NF-water to a final volume of 25 μl. The amplification for both assays were similar, and consisted of 50 °C for 2 min, 95 °C for 10 min followed by 45 cycles of denaturation at 95 °C for 15 s and annealing and extension at 58 °C for 1 min in the *Entamoeba*-assay and at 60 °C for 1 min in the *Giardia*-*Cryptosporidium*-*Dientamoeba*-assay. All DNA templates were analysed undiluted and diluted 1/10 on a 7500 fast real-time PCR system (Applied Biosystems).

### *Blastocystis* barcoding

*Blastocystis* was initially detected and subtyped by the *Blastocystis* barcoding method previously described, using primers RD5 (5′-ATC TGG TTG ATC CTG CCA GT-3′) and BhRDr (5′-GAG CTT TTT AAC TGC AAC AAC G-3′) [29]. One μl DNA was used in a reaction mixture of 20 μl Biomix (Bioline, London, UK), each primer at 250 nM, and NF-water to a total volume of 40 μl. Amplification included an initial step at 94 °C for 5 min followed by 30 cycles of denaturation, annealing and extension at 94 °C, 59 and 72 °C (1 min each) and was completed by a final extension at 72 °C for 2 min. All negative samples were run an additional time with the DNA diluted 1/10 before considered negative. Positive PCR products were purified using illustra MicroSpin S-300 HR columns (GE Healthcare, Little Chalfont, UK). Purified PCR products were sequenced using the primer BhRDr, ABI BigDye terminator kit version 1.1 and an ABI 3130xl Genetic Analyzer (Applied Biosystems). Sequencing chromatograms were analysed in the software Chromas Lite version 2.1.1 (Technelysium, Brisbane, Australia). Subtypes were identified by BLAST searches at the National Center for Biotechnology Information (NCBI) determining the exact match or closest similarity to *Blastocystis* sequences previously deposited in GenBank. The nomenclature for subtypes presented by Stensvold et al. [12] was used.

### qPCR assay to detect *Blastocystis* sp.

To increase the sensitivity of *Blastocystis* detection, all samples negative by the initial barcoding PCR were analysed with a qPCR assay described by Stensvold et al. [30] using primers Blasto FWD F5 (5′-GGT CCG GTG AAC ACT TTG GAT TT-3′) and Blasto R F2 (5′-CCT ACG GAA ACC TTG TTA CGA CTT CA-3′) with the *Blastocystis* probe FAM-5′-TCG TGT AAA TCT TAC CAT TTA GAG GA-3′-MGBNFQ). The reaction mixture consisted of 5 μl DNA template, 12.5 μl TaqMan Universal PCR Master Mix (Applied Biosystems), each primer at 900 nM, hydrolysis probe at 110 nM, and NF-water to a final volume of 25 μl. DNA templates were analysed in dilutions 1/10 and 1/50. The amplification consisted of 50 °C for 2 min, 95 °C for 10 min followed by 45 cycles of 95 °C for 15 s and 60 °C for 1 min.

### *Giardia* assemblage typing

*Giardia* identified by qPCR were assigned to genetic assemblages by conventional PCR, assay 4E1-HP developed by Vanni et al. [31], with specific primers for assemblage A (forward 5′-AAA GAG ATA GTT CGC GAT GTC-3′, reverse 5′-ATT AAC AAA CAG GGA GAC GTA TG-3′) and assemblage B (forward 5′-GAA GTC ATC TCT GGG GCA AG-3′, reverse 5′-GAA GTC TAG ATA AAC GTG TCG G-3′). Originally presented as a duplex assay we used the primer pairs in two separate reactions, which in our hands was more sensitive for mixed infections. An increase in primer concentration from 200 nM to 400 nM yielded easier to read PCR products. Both reaction mixtures contained 2 μl of DNA, 25 μl of BioMix (Bioline), each of the respective primers at 400 nM, and NF-water to a final volume of 50 μl. Amplification was performed on a conventional thermocycler (MyCycler, BioRad Laboratories, Solna, Sweden) with an initial step of 94 °C for 5 min followed by 40 cycles of 94, 56 and 72 °C for 30 s each, completed by a final extension at 72 °C for 7 min. Amplification products were visualized on 1.5 % agarose gels stained with ethidium bromide. Negative samples were analysed with DNA diluted 1/10 to decrease the influence of inhibitors. Remaining negative samples were further examined using NanoDrop (Fisher Scientific, Sweden) for DNA concentration and protein-DNA ratio. A majority of these samples was shown to have a low DNA concentration and were then run with a higher quantity of DNA, 5 μl.

### PCR inhibition control

For control of PCR inhibitors we used a linearized plasmid with a 720 bp insert of *Drosophila melanogaster* ALKcDNA (Christina Lorén, Umeå Center for Molecular Pathogenesis, Umeå, Sweden) in the vector pcDNA3 (Invitrogen, Life Technologies Europe, Stockholm, Sweden). This internal positive control (IPC) was added to all undiluted and diluted 1/10 DNA templates with 125 copies per reaction and was detected by a previously described qPCR assay [23]. The expected cycle threshold (Ct) value was 29,3 and Ct values above 32.5 were considered as signs of relative inhibition [23].

### Statistical analysis

Statistical analysis was performed using the software PASW version 18 (SPSS Inc, Chicago, IL, USA). Pearsons *χ*^2^  test was used for group comparisons of proportions of samples that were positive or negative by microscopy or PCR assays. qPCR Ct values, with higher values indicating lower amounts of target DNA, were compared with independent sample *t*-test.

## Results

### Patients and samples

Stool samples from 174 patients visiting the Jambiani Health Clinic were examined. Among the children less than ten years of age 55/108 (50.9 %) had diarrhoea. Sampling of participants aged ≥ ten years was mainly performed on patients with diarrhoea (62/66) but also included a few patients without diarrhoea (4/66). Non-diarrhoeic patients had other health problems, most commonly respiratory symptoms or fever. The participants were subdivided into age groups (with the number of individuals in each group in parenthesis): 0 < 2 (35), 2 < 6 (35), 6 < 10 (38), 10 < 15 (36), and 15–71 years of age (30). In total, 94 were male (54 %) and 80 (46 %) were female.

### Intestinal parasites identified by PCR-based methods and microscopy after FECT

**Fig. 1:**
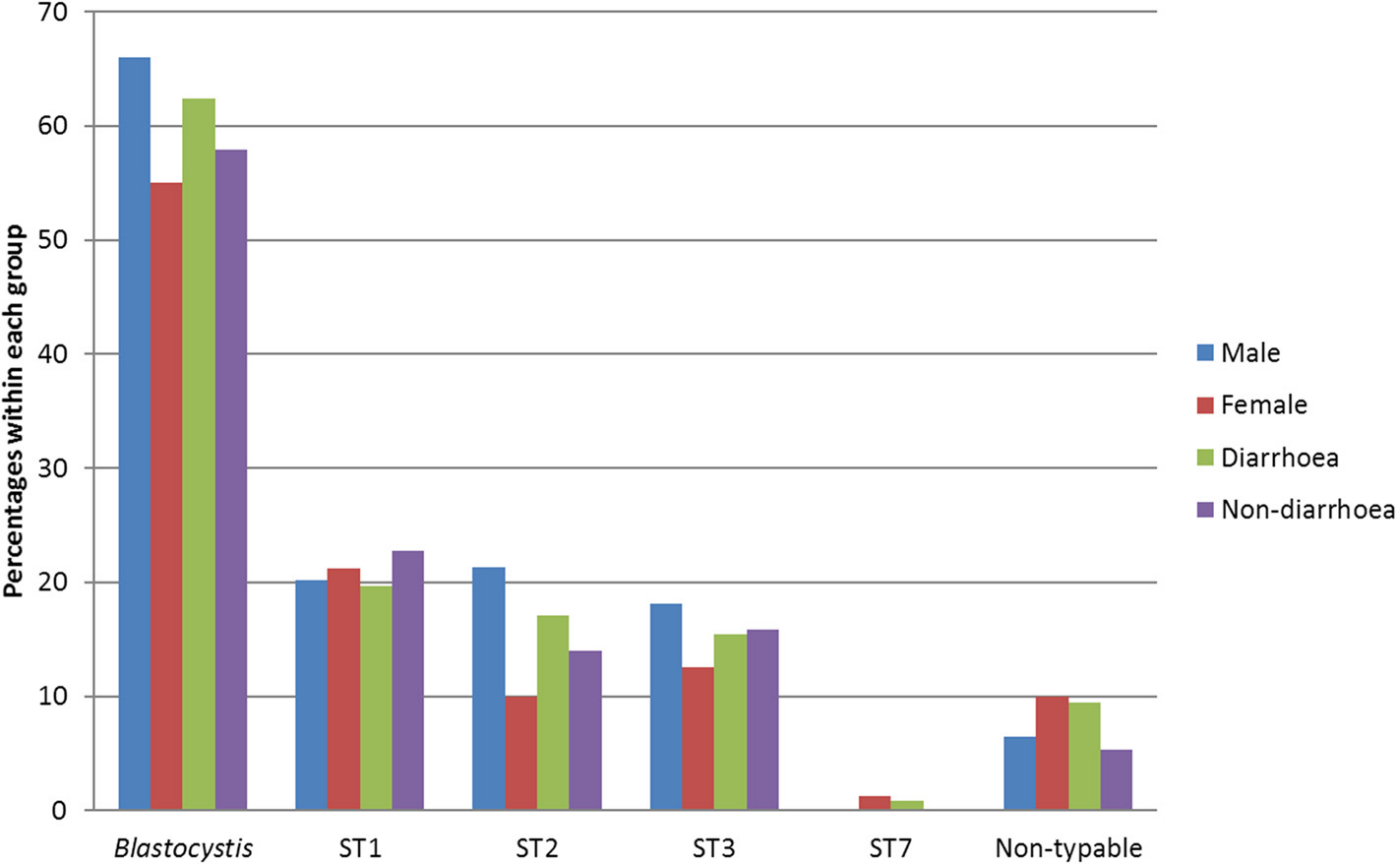
Prevalence of *Blastocystis* sp. and genetic subtypes in males and females and in cases with and without diarrhoea

Summarizing all methods used, intestinal parasites were detected in 85.1 % of the 174 patients (Table 1). Infection with more than one parasite was a common finding. Two, three, four and five parasites were detected in 29.3, 22.4, 2.9 and 1.1 % of the patients, respectively. A single organism was found in only 29.3 % of the patient samples. Using PCR-based methods *Blastocystis* sp. and *Giardia intestinalis* were the most prevalent species, found in 60.9 % (106/174) and 53.4 % (93/174) of the patients, respectively, followed by *Dientamoeba fragilis* in 16.7 % (29/174) (Fig. 1). *Entamoeba dispar* and *Entamoeba histolytica* were found in few samples (Table 1). *Cryptosporidium* spp. was only detected in two patients, both of whom were less than two years old. In 20 samples the initial PCR reaction for the internal control IPC was inhibited but no inhibition was noted after 1/10 dilution of extracted DNA*.*

**Table 1:** Intestinal parasites detected in patients in Zanzibar

	PCR (% positive patients)	Microscopy (% positive patients)
*Blastocystis* sp.	60.9	20.1
*Giardia intestinalis*	53.4	13.8
*Entamoeba coli*	na	21.3
*Dientamoeba fragilis*	16.7	na
*Trichuris trichiura*	na	6.9
*Entamoeba dispar*	5.7	1.7^a^
*Endolimax nana*	na	2.9
*Cryptosporidium* spp.	1.1	0.6
*Chilomastix mesnili*	na	1.1
*Ascaris lumbricoides*	na	0.6
*Entamoeba histolytica*	0.6	0

*Abbreviation*: na, the method was not used for detection

^a^ *Entamoeba histolytica* and *E. dispar* can not be separated by microscopy but subsequent PCR identified *E. dispar*

By microscopy of faecal samples after FECT *Entamoeba coli* was the most common finding, present in 21.3 % of the samples, followed by *Blastocystis* sp. and *Giardia intestinalis* in 20.1 and 13.8 %, respectively. The presence of helminths in the stool samples was only investigated by microscopy, no PCR-based methods were used. *Trichuris trichiura* was the most common helminthic species with a prevalence of 6.9 %. *Ascaris lumbricoides* was only found in one patient (Table 1).

In all species for which both methods were used PCR was more sensitive than identification with microscopy. Six cases of *Blastocystis* sp. detected by microscopy were negative by the initial barcoding PCR assay. Subsequent qPCR for *Blastocystis* was positive in four out of these six cases (with Ct values between 25.6 and 33.1). Overall, the conventional PCR used for *Blastocystis* was more than twice as sensitive as the identification with microscopy - and combined with the qPCR results the molecular tests  - detected three times as many *Blastocystis* as microscopy. All *G. intestinalis*, *E. histolytica/dispar* and *Cryptosporidium* spp. identified by microscopy were also positive by qPCR. The sensitivity to detect *G. intestinalis* by qPCR was more than three times greater than that of microscopy. As a reflection of a possible difference in the number of parasites present in the samples the Ct values in the qPCR were substantially lower (mean 20.8, interquartile range 17.8–24.1) in the samples positive for *Giardia* in microscopy than in microscopy negative samples (mean 30.8, interquartile range 25.2–36.3;  *t*_(56)_ = 8.316,  *P* < 0.001). The one case of *E. histolytica* detected by qPCR (Ct value 35.0) was not detected by microscopy.

When seeking correlations between the most commonly detected parasites, we found that *Blastocystis* was present in 31 of 37 samples where *Entamoeba coli* was detected (*χ*^2^ = 10.32, *df*  = 1, *P* = 0.001) and in 23 of 29 *Dientamoeba* positive samples (*χ*^2^ = 4.94, *df* = 1, *P* = 0.026). We observed no significant correlation between *Blastocystis* and *Giardia* carriage (*χ*^2^ = 1.09, *df* = 1, *P* = 0.297). 

### *Blastocystis* sp. subtyping

The barcoding PCR for *Blastocystis* sp. was positive in 85 cases (48.9 % of the patients) and all these *Blastocystis* were successfully subtyped. By analysing *Blastocystis* negative samples with qPCR an additional 21 cases were found, to a total of 106 positive individuals (60.9 %). Optimised attempts at subtyping the qPCR positive cases were successful in seven out of these 21. In total, the identified subtypes were: ST1 36 (34.0 %), ST2 28 (26.4 %), ST3 27 (25.5 %), ST7 1 (0.9 %), and non-typable 14 (13.2 %). The qPCR amplifies around *c.*115 bp of the SSU-RNA gene. The small size of the product contributes to the high sensitivity of the qPCR but leads to a low ability to discriminate between the *Blastocystis* subtypes. No double peaks in the sequencing chromatograms that would indicate mixed infections with more than one subtype were found with the set of analyses we used. In Table 2 the subtype distribution in this study is presented together with the distributions found in other African countries according to the literature. We found no statistically significant difference in carrier-rates of *Blastocystis* between males (66.0 %) and females (55.0 %). An observed difference in prevalence for ST2 in males (21.3 %) compared to females (10 %) did not reach statistical significance, (*χ*^2^ = 2.62, *df* = 1, *P* = 0.105) (Fig. 1).

**Table 2:** *Blastocystis* sp. subtype distribution in different African countries. Subtypes assigned by sequence-tagged site primers or partial sequencing of the SSU-rRNA gene

Country [reference]	Subtyping method	No. of observations	Subtype^a^									
			1	2	3	4	5	6	7	8	9	Mixed
Egypt [17]	STS^c^	44	8	–	24	–	–	8	4	–	–	–
Egypt [18]	Sequencing	21	4	4	13	–	–	–	–	–	–	1^d^
Egypt [19]	STS^c^	110	15	–	49	–	–	33	13	–	–	10^d^
Egypt [20]	STS^c^	36	6	–	30	–	–	–	–	–	–	–
Liberia [16]	Sequencing	25	7	7	8	3	–	–	–	–	–	5^e^
Libya [16]	Sequencing	38	19	3	15	–	–	–	1	–	–	–
Libya [21]	Sequencing	48	26	13	9	–	–	–	–	–	–	3^d^
Nigeria [16]	Sequencing	22	10	–	9	3	–	–	–	–	–	1^e^
Senegal [9]	Sequencing	93	29	21	51	2	–	–	–	–	–	8^d^
Tanzania [22]	Sequencing	6	1	3	2	–	–	–	–	–	–	–
Tanzania^b^	Sequencing	92	36	28	27	–	–	–	1	–	–	–
Total		535	161	79	237	8	–	41	19	–	–	28

^a^Subtypes denoted according to a consensus denomination [12]

^b^Present study

^c^STS = sequence*-*tagged site primers (specific for each subtype 1–7)

^d^Subtypes involved in mixed infections were resolved and were added to the distribution

^e^Subtypes involved in mixed infections were not resolved

### *Giardia intestinalis* assemblage typing

**Fig. 2:**
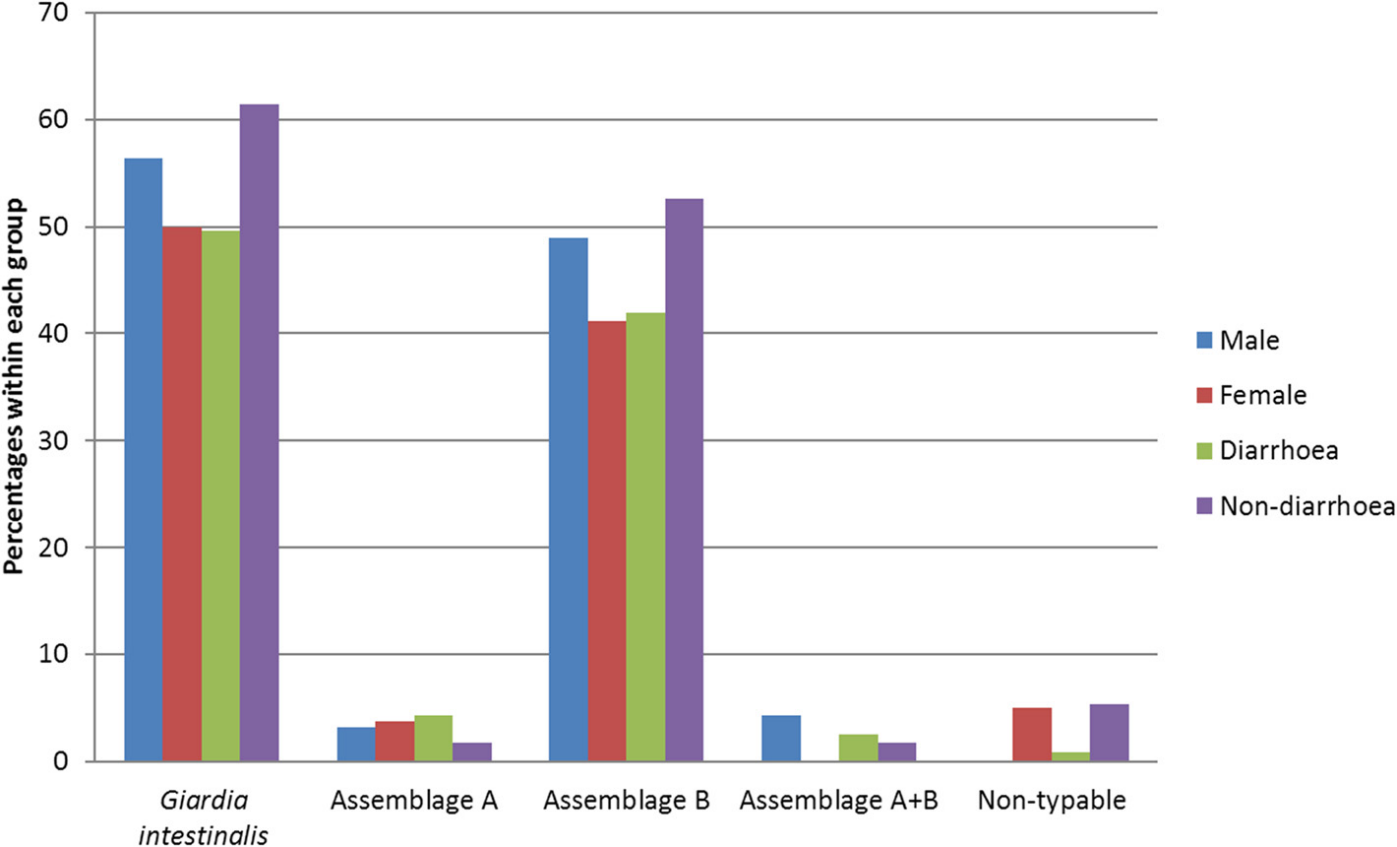
Prevalence of *Giardia intestinalis* and genetic assemblages in males and females and in cases with and without diarrhoea

The 93 *G. intestinalis* identified by qPCR were assigned to genetic assemblages by conventional PCR. A single infection with *G. intestinalis* assemblage B was found in 79 of these 93 samples (85 %), a single infection with assemblage A in six cases (6.5 %), and mixed infections of assemblages A and B in four cases (4.3 %). In four samples (4.3 %) neither of the two tested assemblages were identified despite rigorous attempts at optimising the PCR, these samples were also negative by microscopy and the Ct values in the *Giardia*-qPCR were 33.8–36.2, indicating relatively low amounts of target DNA. No significant difference in the presence of either *G. intestinalis* or *Giardia* assemblage B was noted when males (56.4 and 48.9 % respectively) and females (50.0 and 41.2 % respectively) were compared (Fig. 2). The number of assemblage A cases were too low for valid comparisons.

### Presence of intestinal parasites in relation to age

**Fig. 3:**
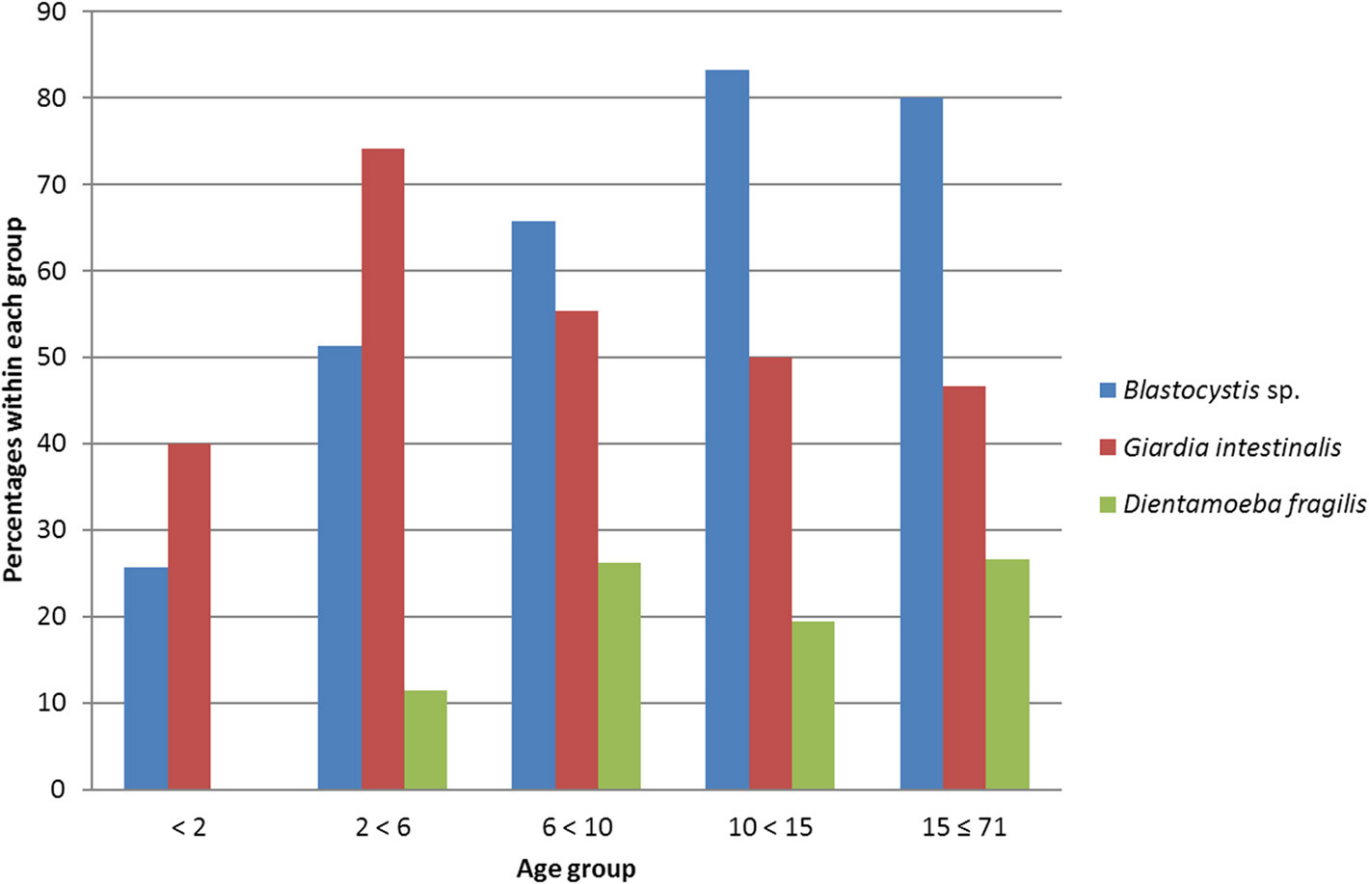
Presence of *Blastocystis* sp., *Giardia intestinalis* and *Dientamoeba fragilis* in stool samples from patients in different age groups in Zanzibar

The prevalence of *Blastocystis* sp., *G. intestinalis* and *D. fragilis* in different age groups is presented in Fig. 3. Detection of *Giardia* was associated with younger age and was more often identified among the youngest individuals compared to those ten years of age or older. The highest prevalence of *Giardia* was seen among children aged two to five years, with a carrier-rate of 74.3 %, which decreased to 46.7 % in the age group 15 years or older (Fig. 3). The presence of *Blastocystis* gradually increased with age. A highly significant difference was seen between both the two youngest age groups where *Blastocystis* was detected in 25.7 and 51.4 % respectively and in teenagers and adults in which around 80 % of the individuals carried *Blastocystis* in their intestine (*χ*^2^ = 24.57, *df* = 1, *P* < 0.001) (Fig. 3). The older age groups preferentially consisted of patients with diarrhoea which may have distorted the results. However, the same relationship to age was seen when only diarrhoeic cases were compared since no correlation between either *Blastocystis* sp. or *G. intestinalis* and the presence or absence of diarrhoea was found in any age group (see below).

### Diarrhoea among patients in relation to identified parasites in stool samples

By comparing carrier-rates in cases with and without diarrhoea we found no statistically significant correlation between diarrhoea and the presence of intestinal parasites or the number of parasites infecting each individual. Furthermore, there were no significant differences in carrier-rates in cases with diarrhoea and without diarrhoea for *Blastocystis* sp. (62.4 *vs* 57.9 %); *Blastocystis* subtypes ST1 (19.7 *vs* 22.8 %), ST2 (17.1 *vs* 14.0 %), ST3 (15.4 *vs* 15.8 %) (Fig. 1); *G. intestinalis* (49.6 *vs* 61.4 %); *Giardia* assemblage B (41.9 *vs* 52.6 %) (Fig. 2); *D. fragilis* (14.5 *vs* 21.1 %); or *E. coli* (20.5 *vs* 22.8 %). This held true not only for the whole study population but also for each age group and gender separately analysed (data not shown). The detected numbers of the other parasite species, including *Giardia* assemblage A, were too low for valid comparisons of symptomatology. No difference in Ct values was noted in the qPCR for *Giardia* when stool samples from patients with or without diarrhoea were compared (mean Ct values 28.2 and 28.5, respectively).

## Discussion

In this study we investigated the occurrence of parasites and the genetic diversity of intestinal protozoa in the stools of individuals seeking community health care in a village in Zanzibar, Tanzania.

### Prevalence of intestinal parasites using microscopy and PCR-based methods

We found a high prevalence of parasites in the stool samples of the 174 participants, 85.1 % harboured one or more parasites. The high prevalence compared to many other studies can possibly be related to the significantly higher detection rate observed by PCR compared to microscopy. The two most commonly found parasites, *Blastocystis* sp. and *G. intestinalis* were detected three times more often by qPCR than by microscopy. An increased sensitivity of molecular detection methods has also been shown by others [32–34]. The detection rate with microscopy would probably have increased if we had collected three samples instead of one. The sensitivity of investigating one individual stool sample by FECT and microscopy, compared to an examination of three stool samples, has been shown to be around 75 % for intestinal parasites in general [35] and *G. intestinalis* in particular [36]. Taking that into account qPCR would still be vastly more sensitive. The higher qPCR Ct values for *Giardia* found in samples not detected by microscopy may indicate that the increased detection rate in large is due to detection of low pathogen loads in the sample. Only one case of *Entamoeba histolytica* was found by qPCR. In this sample, no cysts of *E. histolytica/dispar* were found by microscopy and the Ct value in the qPCR assay was 35.0, indicating that a low number of parasites may have been present in the sample. This discrepancy in detection rates between PCR-based methods and microscopy combined with the relative quantification derived from qPCR-assays could be of interest for further studies of the association between gastro-intestinal symptoms and the presence of unicellular eukaryotes in the intestine.

In a previous study from northwest Zanzibar investigating gastro-intestinal pathogens in children aged two to 59 months, including *Cryptosporidium* as the only parasite, Cryptosporidia were a common finding [37]. This is in contrast to the rare occurrence of *Cryptosporidium* in our study, with only two detected cases. The difference may relate to local geographic differences, the occurrence of a local outbreak, selection of study participants (fever and diarrhoea were inclusion criteria for the symptomatic group in their study), or seasonality of the infections. Similarly, few cases of helminth infections were detected in our study. Infections with the soil-transmitted helminths *Ascaris lumbricoides*, *Trichuris trichiura* and hookworms are globally among the most common human infections and was also described as a major public health problem in Zanzibar in the early 1990s [38]. However, in 1994 a National Helminth Control Programme was initiated in school-aged children in Zanzibar [39] which may explain the few helminth infections detected.

### Prevalence and subtype distribution of *Blastocystis* sp.

*Blastocystis* was the most prevalent parasite in this study, found in 60.9 % of the study participants, with a similar prevalence in patients with and without diarrhoea (Fig. 1).

Another study from sub-Saharan Africa by El Safadi et al. [9], detecting *Blastocystis* in children in Senegal by using the barcoding method [29] and a qPCR assay developed by Poirier et al. [40], found a *Blastocystis* prevalence of 100 % in the studied population of both symptomatic and asymptomatic children aged six to ten years old. Conflicting results regarding the incidence of *Blastocystis* in different age groups can be found in the literature [41–43]. This can in part be explained by study design, where only children or only adults have been examined, or when relatively few *Blastocystis* have been detected by conventional techniques. In a large study from Brazil Cabrine-Santos et al. [42] showed that the prevalence of *Blastocystis* in children was significantly higher above the age of six. This is in agreement with our findings where the prevalence of *Blastocystis* was significantly lower in children below the age of six than in children older than ten years and adults, in which around 80 % of the patients carried *Blastocystis* (Fig. 3). However, our results from individuals over ten years old should be substantiated by a larger study because of a small number of non-diarrhoeic controls (6.1 %) and the wide age spread among the 30 individuals that constitute the oldest age group 15–71 years of age.

In the subtype analysis, ST1 was the most commonly found subtype, followed by ST2 and ST3, with a rather even distribution between these three subtypes. No mixed infections were detected. A higher prevalence of mixed infections may be possible to identify with other methods, as has been suggested in a recent study in which a high percentage of mixed infections, especially involving ST1, was found in stool samples from healthy humans with the use of a ST-specific nested PCR [44]. Globally, ST3 is the most commonly detected subtype followed by ST1 [16]. This pattern is also seen in the compiled studies from Africa, shown in Table 2. A predominance of ST1 has been reported in some studies from Africa [16, 21], Asia [45, 46] and South America [47]. ST1 was also found among non-human primates in the northwest region of Tanzania [22]. ST4, which is common in Europe accounting for 24.2 % of studied *Blastocystis* isolates [16, 48], was completely absent in our study. Low prevalence or complete absence of ST4 has repeatedly been found in studies from Asia and Africa [16]. How the different *Blastocystis* subtypes are spread and why ST4 is absent in certain areas of the world is not known. The authors of a study on *Blastocystis* subtype prevalence in the UK, Liberia, Libya and Nigeria [16] point out that ST4 seems rare in regions where ST1 is the dominant subtype and in regions with mainly Muslim populations, both of which are true for our study. These geographical differences in *Blastocystis* subtype prevalence prompts the need to investigate the molecular epidemiology of *Blastocystis* in regions and populations not previously investigated.

Our findings, with a high prevalence of *Blastocystis,* an increasing prevalence with age, and the absence of mixed subtype infections, might indicate a gradual establishment of *Blastocystis* carriage over time. Further longitudinal studies on the length and stability of *Blastocystis* subtype carriership are needed to determine if the high prevalence in older children and adults is caused by frequent reinfections or, once acquired, stable long term colonisation, and if so, its role in the human intestinal microbiota. We found no correlation between diarrhoea and any specific *Blastocystis* subtype. Research on the pathogenic potential of different subtypes is ongoing and some studies have indicated certain subtypes as more likely to be pathogenic (reviewed in Roberts et al. [10]), but to date, no clear distinction between pathogenic and nonpathogenic subtypes have been made.

### Prevalence and assemblage distribution of *Giardia intestinalis*

*Giardia intestinalis* is a common cause of diarrhoea worldwide, with approximately 280 million cases of giardiasis annually [49]. The highest prevalence of giardiasis is seen in tropical and subtropical low-income countries. *Giardia* is considered a significant cause of diarrhoea and nutritional disorders in these countries and is included in the WHO Neglected Diseases Initiative with the aim to improve health and socio-economic development [50]. In endemic areas, *Giardia* is especially prevalent among children and asymptomatic infections are common [51–53]. In our study the presence of *Giardia* was high in all age groups investigated but was most prevalent in children two to five years of age, where the parasite was detected in as many as 74 %.

Isoenzyme electrophoresis investigations on *Giardia* isolates have found seven genetic subgroups, named assemblage A to G, where A and B predominately infect humans [54]. Molecular characterisation has shown that the genomes of assemblage A and B share 77 % identity in protein coding regions, which suggest that they may represent two separate species [55]. The high detection rate of *Giardia* found by qPCR in our study was confirmed by the conventional PCR assays for *G. intestinalis* assemblage A and B that gave positive results for 89 of 94 qPCR positive cases, 85 % of which were assemblage B. The lack of assemblage identification in four samples positive in qPCR for *Giardia* may indicate that these *Giardia* belong to another assemblage. However, the samples were negative by microscopy and had high Ct values in the qPCR and we think that a more plausible explanation is a low number of organisms present in these samples. The prevalence of *G. intestinalis* found in our study*,* 53.4 % by qPCR and 13.8 % by microscopy, and a dominance of assemblage B, is similar to findings in a study of intestinal parasites among children under five years of age in Rwanda [56], where the authors found *G. intestinalis* by qPCR in 59.7 %, by microscopy in 19.8 % and assemblage B in 86 % of the *Giardia* positive cases. A predominance of assemblage B has also been found in Pemba Island, Zanzibar, Tanzania [57], as well as in studies from other African countries such as Guinea-Bissau [58], Morocco [59], Algeria [60], Uganda [53], Côte d’Ivoire [61] and Egypt [62–64]. Assemblage A was the most prevalent genotype in another study from Egypt [65] and in one study from Ethiopia [66]. Studies regarding the association of assemblage A and B to symptomatic infections have reached discordant conclusions [67]. Our data included too few cases of assemblage A infections to reliably investigate any differences in pathogenic potential between the assemblages. Larger studies are needed to determine if the non-dominant assemblage in *Giardia* endemic regions has a different virulence to the dominant assemblage.

### The role of *Blastocystis* sp. and *Giardia intestinalis* in causing diarrhoea

Both *Blastocystis* and *Giardia* were common findings, among both diarrhoeic and non-diarrhoeic individuals. This is an interesting background to the debate regarding the pathogenicity of *Blastocystis* and the role intestinal parasites have in causing gastro-intestinal symptoms. In a case-control study on diarrhoea among children under five years in Tanzania 52.2 % of asymptomatic individuals carried one or more enteropathogens. The authors found that *Shigella* was the only detected organism statistically linked to diarrhoea - *Salmonella*, ETEC, EPEC, EAEC and *Giardia* were not [68]. Similarly, Platts-Mills et al. found an association between diarrhoea and detection of *Shigella/*enteroinvasive *Escherichia coli*, rotavirus and astrovirus in Tanzanian infants; however, only when the quantity of the organisms was taken in consideration [69]. In our study, Ct values used as a measure of abundance were indistinguishable between patients carrying *Giardia* with or without diarrhoea. In western countries patients are treated both in symptomatic and asymptomatic *Giardia* cases, the latter to prevent nutrient deficiencies. In highly endemic areas, such as Zanzibar, treatment is not recommended in asymptomatic cases because of frequent reinfections. This is not unproblematic, however, since recent research have shown that giardiasis in childhood is associated with malnutrition, stunted growth and impaired cognitive development regardless of the presence of diarrhoea [56, 70, 71]. *Blastocystis* sp. on the other hand, is common in western high income countries (7–20 % prevalence) where other intestinal parasites are quite rare. Because of the uncertain pathogenicity, it is mainly treated in prolonged cases of gastrointestinal disturbances where other possible causes are ruled out. In our study *Giardia* was most prevalent among children two to five years of age, in agreement with several reports from developing countries [53, 56, 71]. An immunity response, which can be individually variable and influenced by nutritional status, genetic factors, and repeated exposure to *Giardia*, has been suggested to contribute to lower detection rates of *Giardia* seen in older children and also as an explanation to asymptomatic infections [67]. Although the examined population is too small to draw any firm conclusions, the different age distributions of *Giardia* and *Blastocystis* found in our study with increasing prevalence of *Blastocystis* with increasing age may indicate that the two parasites interact differently with the nonspecific and specific immune defence of the host. More extensive case-controlled studies of different *Blastocystis* subtypes in patients with gastrointestinal diseases, as well as longitudinal studies to determine the clinical impact of primary infections, are required to elucidate the role of *Blastocystis* in gastro-intestinal health and disease.

## Conclusions

Carriage of intestinal parasites was very common in the studied population in Zanzibar. Compared to traditional detection with microscopy we found PCR-based methods to be vastly more sensitive and these should be used in further epidemiological studies. *Blastocystis* was the most common parasite, and subtype analysis revealed ST1-3 to be common. ST4, a subtype common in Europe, was completely absent in our study, further corroborating the geographical differences in subtype distributions previously reported. We found that *Blastocystis* and *Giardia* had different age distributions, possibly indicating differences in transmission routes, immunity, and/or other host factors impacting these two species in this region.
